# Cold stress changes antioxidant defense system, phenylpropanoid contents and expression of genes involved in their biosynthesis in *Ocimum basilicum* L.

**DOI:** 10.1038/s41598-020-62090-z

**Published:** 2020-03-24

**Authors:** Ramin Rezaie, Babak Abdollahi Mandoulakani, Mohammad Fattahi

**Affiliations:** 1 Department of Plant Production and Genetics, Faculty of Agriculture, Urmia University, Urmia, Iran; 2 Department of Agricultural Biotechnology, Institute of Biotechnology, Urmia University, Urmia, Iran; 3 Department of Horticultural Science, Faculty of Agriculture, Urmia University, Urmia, Iran

**Keywords:** Gene expression, Abiotic

## Abstract

Environmental stresses might alter the activity of antioxidant defense system and both quantity and quality of the essential oil constituents in aromatic plants. In the current study, a greenhouse experiment was designed to assess the influence of cold stress on total phenolic (TPC) and flavonoid contents (TFC), DPPH radical scavenging, antioxidant and phenylalanine ammonia-lyase (PAL) enzymes activity and content of phenylpropanoid compounds in *Ocimum basilicum* L. The genes expression levels of chavicol *O*-methyl transferase (*CVOMT*), cinnamate 4-hydroxylase (*C4H*), eugenol synthase 1 (*EGS1*) and eugenol *O*-methyl transferase (*EOMT*) were also investigated. Results revealed the highest TPC, TFC and DPPH at 4 °C for 12 h. Positive significant correlation was observed between TFC and DPPH, as well as TPC and PAL enzyme activity. The highest activity of superoxide dismutase and guaiacol peroxidase was recorded in 4 °C for 48 h, while this treatment caused the highest reduction in the activities of ascorbate peroxidase and catalase. In plants exposed to 10 °C for 48 h, the contents of methyleugenol and methylchavicol was positively associated with the expression levels of *EGS1 and EOMT*. A positive correlation was also found between *C4H* expression and eugenol, methyleugenol and methylchavicol contents under 4 °C for 12 h.

## Introduction

**Figure 1:**
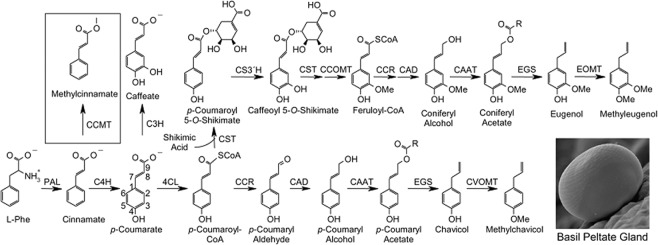
Methylchavicol and methyleugenol biosynthesis pathway in basil glandular trichomes, enzyme abbreviations: PAL, phenylalanine ammonia lyase; CCMT, *p*-coumarate/cinnamate carboxyl methyltransferase; C4H, cinnamate 4-hydroxylase; 4CL, 4-coumarate:CoA ligase; C3H, *p*-coumarate 3-hydroxylase; CST, *p*-coumaroyl shikimate transferase; CS3′H, *p*-coumaroyl 5-*O*-shikimate 3′-hydroxylase; CCOMT, caffeoyl-CoA *O*-methyltransferase; CCR, cinnamoyl-CoA reductase; CAD, cinnamyl alcohol dehydrogenase; CAAT, coniferyl alcohol acetyl transferase; EGS, eugenol (and chavicol) synthase; EOMT, eugenol *O*-methyltransferase; CVOMT, chavicol *O*-methyltransferase^8^.

*Ocimum* genus, belonging to family Lamiaceae, is a source of economically valuable aromatic oils with medicinal properties. This genus possesses an extensive intra- and inter-specific genetic diversification including 65 to more than 150 species spread throughout the world^1^. *Ocimum sanctum* and *O. basilicum* (sweet basil), two high priority species in this genus, are extensively used for their pharmaceutical and industrial importance. *O. basilicum* L. (2n = 4x = 48), originally from India, Iran and Afghanistan contains multiple terpenoid and phenylpropanoid compounds which are used in medicinal and perfume industries^2^. This compounds mostly produces in glandular trichomes on the leaf surface^3^. The most important compounds related to fragrance property are linalool, 1,8 cineole, methylchavicol (estragole) and eugenol. Basil essential oil (EO) has anticancer and hypoglycemic tuberculosis properties^4,5^. Also cytotoxic property of ethanolic extract of basil containing eugenol has been reported on human laryngeal cancer cells^5^. Terpenoids and phenylpropanoids such as eugenol, methylchavicol, methyleugenol and cinnamate are the main constitutes of basil EO^4^. The major phenylpropanoid components of basil are methylchavicol and methyleugenol. These compounds are extensively used in perfumes, aroma therapy, food and medicinal industries. Methylchavicol stimulates liver regeneration, shows hypothermic and DNA binding and spasmolytic activities. Phenylpropanoid compounds is produced through shikimate pathway. Phenylalanine ammonia-lyase (PAL), involved in the initial step of phenylpropanoid metabolism, deaminates phenylalanine to generate trans-cinnamic acid and ammonia^3^. Cinnamate 4-hydroxylase (C4H), a cytochrome P450-dependent monooxygenase, catalyzes the hydroxylation of cinnamate to produce 4-coumarate (*p*-coumarate). At the end of this pathway, eugenol, methyleugenol and methylchavicol are produced by eugenol synthase 1 (EGS1), eugenol *O*-methyl transferase (EOMT) and chavicole *O*-methyl transferase (CVOMT), respectively^6–8^ (Fig. 1).

Excessive generation of reactive oxygen species (ROS) under cold stress can causes sever oxidative damage in plants. The antioxidant defense mechanisms have been developed in plants to diminish the deleterious effects of ROS on plant cells. Antioxidant machinery includes antioxidant enzymes like superoxide dismutase (SOD) and peroxidases and several non-enzymatic antioxidants. Non-enzymatic antioxidants such as phenolic compounds (Phenolic acids and flavonoids) can trap and scavenge free radicals^9,10^. Also, environmental stresses and different ecological conditions substantially affect the EO content and constituents in medicinal plants. Accumulation of the plant secondary metabolites and the expression profile of genes responsible for their biosynthesis, are forcefully correlated with the growth conditions. It has been reported that drought stress may alter the EO yield and composition in plants and generally produces the higher levels of secondary metabolites^11–13^. An increase in EO percentage and compositions have been noted in both *O. basilicum* and *O. americanum* plants subjected to drought stress^14^. In sweet basil plants exposed to drought stress, methylchavicol and methyleugenol accumulation was commensurate with the expression levels of *CVOMT* and *EOMT* genes^15^. The higher level of methylchavicol content was also detected in sweet basil under sever water stress which was positively correlated with transcriptional levels of *CVOMT*^16^. In *O. basilicum* plants treated with drought stress, linalool synthase expression was directly associated with the linalool production^17^. Nasrollahi *et al*.^12^ demonstrated that the use of controlled drought stress upregulates the expression of sequalene and b-amyrin synthases involved in saponins biosynthesis and directly strengthens the accumulation of secondary metabolites, including glycyrrhizin, in liquorice plants. Undeviating affirmative associations between germacrene D and γ-cadinene accumulation and the expression of germacrene D (*GDS*) and γ-cadinene synthases (*CDS*) have been indicated in *O. basilicum* plants exposed to cold stress^18^.

To date, the influence of cold stress on phenylpropanoid components and expression of their biosynthetic genes has not been investigated in *O. basilicum*. Due to the presence of valuable phenylpropanoids with pharmaceutical activities in *O. basilicum*, study the genes expression profiles responsible for phenylpropanoids production and their correlation with the accumulation of corresponded compounds under various environmental conditions may be of interest for the biomedical industry and accelerate the improvement of economically elite basil varieties through genetic engineering techniques. Therefore, the current experiment was designed to investigate the effect of cold stress on the antioxidant defense system, PAL enzyme activity, the genes expression of *C4H*, *EGS1*, *EOMT* and *CVOMT* and phenylpropanoid contents in basil.

## Materials and Methods

### Plant material and cold treatment conditions

Seeds of *O. basilicum* var. keshkeni luvelou, kindly supplied by Prof. A. Hassani (Urmia University, Iran), were grown in plastic pots (diameter: 31 and height: 27 cm) containing a mixture of soil (clay loam, pH = 8.1): sand (3:1), under natural light conditions in greenhouse. The seedlings of each pot were then thinned to ten and 35-days old plants (6–8 leaf stage) were exposed to cold stress in a climate chamber with a 16 h light/8 h dark photoperiod at 4 and 10 °C. Control plants were retained at 22 °C. Leaf samples were collected from cold-treated and control plants 12, 24 and 48 h after treatment^18–20^ application and frozen at −80 °C for antioxidant-related assessments and real time PCR analysis. Cold treatments are referred as T10–12, T10–24, T10–48, T4–12, T4–24 and T4–48. All treated plants were returned to the greenhouse at normal temperature. Whole 60-days old plants were collected at flowering stage for essential oil analysis.

### DPPH radical scavenging assay

Dried leaves were finely ground with mortar and pestle. Then, one g of the well prepared powder were added to the vials containing methanol solvent (80%) and treated at ultrasonic temperatures (45 °C) for 30 min. Data was captured using an ultrasonic water bath (750 W, 50/60 Hz, E 120 H Elmasonic) (Singem, Germany). The prepared extracts were filtered (HPLC 0.45 um porosity) into neat vials and used to measure total phenol, total flavonoid and DPPH%^21^.

To assay free radical scavenging activity^22^, 50 µl of the prepared extract with respect to the does explained above was added to 950 µl of 6 × 10^−5^ mol. l^−1^ (free radical, 95%) in methanol. The solution was precisely shocked and left at room temperature for 15 min. The absorption was then measured at 517 nm and the inhibition percentage of the DPPH radical was appraised as follows:

$${\rm{DPPHsc}}\, \% =\frac{{({\rm{Abs}}{\rm{control}})}_{t=15min}-{({\rm{Abs}}{\rm{sample}})}_{t=15min}}{{({\rm{Abs}}{\rm{control}})}_{t=15min}}\times 100$$

where Abs control and sample is the absorbance of control (included all reagents except the extract) and mixture (extract included) reactions, respectively.

### Determination of total phenolic and flavonoid contents

Total phenolic content (TPC) of the ethanolic extracts, was measured using the Folin-Ciocalteu colorimetric method^21,23^. 50 µl of the prepared extract with 600 µl of Folin-Ciocalteu reagent (10%) thoroughly mixed with 90 µl of distilled water and remained for 10 min. The reaction was neutralized with 480 µl of sodium carbonate and color change of the extracts was recorded after 2 h at 765 nm by UV-2100 spectrophotometer (UNICO, China). The concentration of phenolic compounds was stated as mg gallic acid equivalents (GAE) per g dry matter basis.

A colorimetric assay^23^ was used to quantify total flavonoid content (TFC). 100 µl of the prepared extracts was added to a 15 ml tube containing 2 ml of distilled water. Subsequently, 150 µl of 5% sodium nitrite was added and the mixture left at room temperature for 5 min. Then, 300 µl of 10% aluminum chloride (AlCl_3_. 6 H_2_O) was added. After 6 min, 1 ml of 1 mol. l^−1^ sodium hydroxide was added and finally the volume of the mixture was brought to 5 ml by distilled water. The absorbance of the solution was immediately assayed at 380 nm and TFC was stated as quercetin equivalents using a standard curve created from authentic quercetin.

### Antioxidant and PAL enzyme assays

Frozen leaf samples were homogenized with ice-cold 50 mM buffer (Tris-HCl, pH 7.5) using a mortar and pestle in an ice bath and centrifuged two times at 12000 rpm at 4 °C for 20 min, and at 9000 rpm at 4 °C for 15 min. Finally, supernatant was used for enzyme assays. The activity of guaiacol peroxidase (GPX) was determined by spectrophotometer with guaiacol as the substrate in a total volume of 2 mL. The reaction mixture contained 50 mM phosphate buffer (pH 6.6), guaiacol (1%), H_2_O_2_ (0.3%) and enzyme extract (50 µl). Increase in the absorbance, due to the guaiacol oxidation, was measured at 470 nm for 1 min^24^. Catalase (CAT) activity was measured by spectrophotometer in a reaction mixture containing 50 mM phosphate buffer (pH 7.0), 5 mM H_2_O_2_ (3%) in a total volume of 2.8 mL and 20 µl of enzyme extract. The H_2_O_2_ decomposition was followed by the decrement in absorbance at 240 nm^25^.

A reaction mixture of 2.5 mL (50 mM phosphate buffer, pH = 7.0, 5 mM H_2_O_2_ and 100 µl of enzyme extract) was used to measure the activity of ascorbate peroxidase (APX) by spectrophotometer method^24^. The oxidation rate of APX was assayed by the decline in absorbance at 290 nm. Superoxide dismutase activity was assayed by rate inhibition of nitro blue tetrazolium (NBT) at 560 nm. Specific enzyme activity was stated as Units/g fw and one unit of SOD activity was specified as the amount of the enzyme required for inhibition of NBT reduction by 50%. PAL activity was measured at 290 nm and defined as nm/gr fw.

### Phenylpropanoid isolation and identification

Dried aerial parts of the plants was used to extract EO by conventional hydro distillation for 2 h in a Clevenger. EO compounds were analyzed using gas chromatography/mass spectrometry (GC/MS), Thermo Finnegan Quest Trace Ms pluse, equipped with a HP-5 column (30 m length, 0.25 mm inner diameter, film thickness 0.25 μm). Oven temperature was increased from 40 °C to 160 °C at a rate of 4 °C/min and enhanced again from 160 to 280 with a speed of 5 °C/min. Then was retained at 280 °C for 10 min. Helium was applied as the carrier gas at a flow rate of 1 ml/min and ionization energy 70 eV^18^. The individual compounds were characterized by using different parameters such as time, retention index (RI), mass spectra and their comparison with those of the internal reference in mass spectra library or authentic compounds and with those from the literature^26^. Out of 40 compounds (Supplementary Table S1) characterized in our study, only phenylpropanoids were investigated.

### Isolation of total RNA and cDNA conversion

RNA was isolated from 0.2 g leaf tissues by RNX^TM^-Plus kit (SinaClon, Iran) following the manufacturer’s instructions. The quality and concentration of the RNA were measured using 1% agarose gel electrophoresis and Nanodrop. Removal of residual genomic DNA from the total RNA and cDNAs synthesis were performed using Revert Aid™ First Strand cDNA Synthesis Kit (Thermo Scientific, USA) using the manufacturer’s instructions. Reverse transcriptase minus (-RT) negative and no template controls (NTC), were used during cDNA synthesis to evaluate the RNA samples for genomic DNA and reagent contaminations, respectively. To verify the cDNA synthesis kit, glyceraldehyde 3-phosphate dehydrogenase (*GAPDH*) control RNA (1.3 kb), provided with the kit, was also converted to cDNA and PCR of this cDNA using specific primers revealed a fragment of 496 bp in 1.8% agarose gel (Supplementary Fig. S1).

### Real time PCR analysis

Coding sequences of the *C4H*, *EGS1*, *EOMT* and *CVOMT* genes were downloaded from gene bank (www.ncbi.nlm.nih.gov) and specific primers (Table 1) were designed using Fast PCR 4.0^27^ and Gen runner 3.05 (Hastings Software Inc., Hastings, NY, USA; http://www.generunner.net/) software’s. BLAST of the primers against nucleotide sequences in NCBI was done to validate their specificity. A 20 μl PCR reaction containing 3 μl diluted cDNA (1:2), 1 × PCR buffer (10 mM Tris–HCl, 50 mM KCl, pH = 8.3), 1.5 mM MgCl2, 0.2 μM dNTP, 0.5 unit of Taq DNA polymerase, and 10 pmol of primer was used to optimize the annealing temperature of the genes^18^. The PCR amplification, running in an Eppendorf thermal cycler, consisted of a pre-denaturation step of 2 min at 95 °C, 35 cycles of 40 s at 95 °C, 30 s at 58–60 °C and 50 s at 72 °C, continued by a final extension of 5 min at 72 °C^18^. Amplified products were resolved on 1.8% agarose gel (Supplementary Fig. S1) and visualized with ethidium bromide using a Gel documentation system (Infinity, France). Real-time PCRs were carried out in a volume of 12.5 μl in Rotor-Gene Q (QIAGEN, USA) using Maxima SYBER Green/Fluorescein qPCR Master Mix (Thermo Scientific, USA) following the manufacturer’s instructions. Temperature conditions were as; holding for 10 min at 95 °C and 40 cycles of 95 °C for 15 s, 58 to 60 °C (Table 1) for 30 s and 72 °C for 40s^18^. Three biological replicates for each treatment were used for real-time PCR analysis. Melt curve analysis and 1.8% agarose gel electrophoresis were used to confirm the specificity of the amplicons. Two reference genes, 18s-rRNA and actin, were used for data normalization and the reference gene (actin) showing the cycle of threshold (CT) values near to those of the studied genes was applied for data normalization.

**Table 1:** Accession number, annealing temperature (Ta), sequence and product size of the real time PCR primers used in the current study.

Gene	Accession number	Annealing temperature (Ċ°)	Primer sequences (5′-3′)	Product Size (bp)
*18s rRNA*	AK059783	60	F: CTACGTCCCTGCCCTTTGTACA	65
			R: ACACTTCACCGGACCATTCAA	
*Actin*	AF282624	57	F: GCAGGGATCCACGAGACCC	95
			R: CCCACCATGAGCACCAC	
*CVOMT*	AF435007.1	57	F: ATTGGTCGATGTTGGGGGTG	93
			R: TGTGGTAGGTCAAGAACAGTGC	
*EOMT*	AF435008	57	F: CAAGAGGTGTGCTACTGGCT	88
			R: ACGACTTGGACTAGGGGTGT	
*EGS1*	DQ372812.1	60	F: ACCCATAGCAATCCTTCACTG	85
			R: AGTTGAAGCCTCCACATCGT	
*C4H*	HM990150.1	58	F: GCCAACAACCCCGCTCAATG	119
			R: CCAACGCCGAAGGGGAGGTATC	

*CVOMT*: chavicol *O*-methyl transferase, *EOMT*: eugenol *O*-methyl transferase, *EGS1*: eugenol synthase1, *C4H*: cinnamate 4-hydroxylase.

### Statistical analysis

The experiment was arranged in a completely randomized design with three replications. Analysis of variance was performed to evaluate the effect of cold stress on TPC, TFC, DPPH, studied antioxidant and PAL enzymes activity and the content of phenylpropanoid compounds. Then mean of the treatments were compared by Duncan’s method in SAS program ver. 9.1. Before analysis of variance, normality test of the data and residuals was examined using Kolmogorov-Smirnov test in software MINITAB 19. The results of this test showed that both data and residuals follow the Normal distribution. The correlation between the studied variables was also calculated using the same software. Relative expression of the studied genes in treated over the control plants was measured using ΔΔCT method^28,29^. Analysis of variance and pair-wise comparisons of the means using Duncan’s test were carried out for gene expression data as well.

## Results

### Total phenolic and flavonoid contents and DPPH radical scavenging assay

TPC, TFC and DPPH were significantly (*P* ≤ 0.01) affected by cold treatments (Table 2). The highest TPC was recorded in T10–24 which is not significantly differed from T4–12. The minimum amount of TPC observed in plants grown under normal conditions. The highest TFC and DPPH obtained in T4–12 (Table 3). A positive significant correlation (r = 0.8, *P* ≤ 0.01) was found between TFC and DPPH. The correlation between TPC and DPPH was also positive but not significant.

**Table 2:** Analysis of variance for TPC, TFC, DPPH and antioxidant and PAL enzymes activity under cold stress in *Ocimum basilicum*.

Mean of squares								
Source of variance	TPC	TFC	DPPH	APX	CAT	GPX	SOD	PAL
Cold stress	9.175**	20.205**	0.028**	0.001**	0.0003**	0.000002**	9871.88**	22805.77**
Error	0.886	0.094	0.001	3E-06	0.00004	1E-07	273.09	3.253
Coefficient of variation (%)	7.57	5.3	5.02	3.35	1.27	17.8	3.85	0.39

TPC: total phenolic content, TFC: total flavonoid content, APX: ascorbate peroxidase, GPX: guaiacol peroxidase, SOD: superoxide dismutase, PAL: phenylalanine ammonia-lyase, **means significant at level 0.01 (*P* ≤ 0.01).

**Table 3:** The effect of cold stress treatments on TPC, TFC, DPPH and antioxidant and PAL enzymes activity in *Ocimum basilicum*.

Compounds	Means						
	TN	T10–12	T10–24	T10–48	T4–12	T4–24	T4–48
TPC (mg GAE/g dw)	10.25 c	12.64 b	14.91 a	11.45 bc	14.57 a	12.08 b	11.1 bc
TFC (mg QUE/g dw)	5.28 c	4.23 e	7.32 b	5 cd	10.9 a	3.06 f	4.67 de
DPPH (%)	0.62 b	0.55 d	0.57 cd	0.62 b	0.84 a	0.59 bc	0.62 b
APX (mM/g fw)	0.040 d	0.052 b	0.053 b	0.068 a	0.067 a	0.046 c	0.032 e
CAT (mM/g fw)	0.013 bc	0.017 b	0.012 bc	0.0047c	0.019 b	0.035 a	0.0031 c
GPX (mM/g fw)	0.0015 bc	0.0025 a	0.0008 c	0.0019 b	0.0011 c	0.0010 c	0.0029 a
SOD (U/mg pr)	332 d	413.33 b	469.33 a	473.67 a	372.67 c	460 a	477.33 a
PAL (nm/gr fw)	444.77 d	541.66 a	535.40 b	332.63 f	545.06 a	461.27 c	359.25 e

TPC: total phenolic content, TFC: total flavonoid content, APX: ascorbate peroxidase, GPX: guaiacol peroxidase, SOD: superoxide dismutase, PAL: Phenylalanine ammonia-lyase, TN: untreated plants (normal condition), T10–12, T10–24, T10–48, T4–12, T4–24 and T4–48 refer to the cold-stressed plants cultivated under 12, 24 and 48 h at 4 and 10 °C. Common letters in each row show no significant difference at *P* ≤ 0.01.

### Antioxidant and PAL enzyme activity

The activity of all antioxidant enzymes assayed, as well as PAL were significantly (*P* ≤ 0.01) influenced by cold treatments (Table 2). The highest activity of APX and CAT observed in T10–48 and T4–24, respectively, while GPX and SOD showed the maximum activity in T4–48. The minimum activity of APX and CAT observed in plants exposed to 4 °C for 48 h. The activity of PAL reached the maximum in T4–12. The long-period cold treatments (T10–48 and T4–48) reduced the activity of PAL, significantly (Table 3). Also, a significant positive correlation (r = 0.0.7, *P* ≤ 0.01) was found between PAL (an enzyme involved in the biosynthesis of phenolic compounds) activity and total phenolic compounds.

### Effect of cold stress on phenylpropanoid compounds

In the present study, analysis of variance was performed to assess the effect of cold stress on EO percentage and the content of camphor, borneol, methylchavicol, bornyl acetate, α-cubebene, eugenol, β-cubebene, β-elemene, methyleugenol, cadina-1(10), 4-diene, δ-cadinol, spathulenol, cubenol, epi-α-cadinol and t-muurolol. Cold stress significantly (*P* ≤ 0.01) affected the content of all studied components (Table 4). The highest EO percentage was observed in plants under treatment T4–48. The highest amount of camphor, eugenol, δ-cadinol and cubenol achieved in T10–12 while methyleugenol content reached the maximum at T4–24. The highest amount of methylchavicol and β-elemene achieved under T10–12 and T10–24. Bornyl acetate, epi-α-cadinol and t-muurolol had the highest amount at T10–12 and T4–24 (Table 5).

**Table 4:** Analysis of variance for essential oil percentage and studied phenylpropanoid compounds under cold stress in *Ocimum basilicum*.

Mean of squares								
Source of variance	EoP	Cam	Bor	Bor A	α-Cub	β-Cub	β-Ele	Eug
Cold stress	0.022**	0.034**	0.003**	0.045**	0.00068**	0.017**	0.14**	5.25**
Error	0.0007	0.004	0.00019	0.005	0.0001	0.001	0.0067	0.44
Coefficient of variation **(%)**	3.67	7.72	20.7	14.1	22.58	21.25	7.07	11.53
**Mean of squares**								
**Source of variance**	**MCh**	**MEug**	**δ-Cadi**	**Spat**	**Cadi**	**Cub**	**E-α-c**	**Tmu**
Cold stress	0.904**	0.325**	0.0004**	0.0034**	0.0077**	0.023**	1.523**	0.005**
Error	0.0041	0.0086	0.00004	0.00041	0.0013	0.0029	0.71	0.0001
Coefficient of variation **(%)**	9.014	7.86	29.57	29.47	25.77	9.43	6.28	14.94

EoP: essential oil percentage, Cam: camphor, Bor: borneol, MCh: methylchavicol (estragole), Bor A: bornyl acetate, α-Cub:α-cubebene, Eug: eugenol, β-Cub: β-cubebene, β-Ele: β-elemene, MEug: methyleugenol, Cadi: cadina-1(10),4-diene, δ-cadi: δ-cadinol, Spat: spathulenol, Cub: cubenol, E-α-c: epi-α-cadinol, Tmu: t-muurolol. **Mmeans significant at level 0.01 (*P* ≤ 0.01).

**Table 5:** The effect of cold stress treatments on essential oil (EO) percentage and identified phenylpropanoid compounds in *Ocimum basilicum*.

Compounds	Means						
	TN	T10–12	T10–24	T10–48	T4–12	T4–24	T4–48
EO percentage	0.75 b	0.75 b	0.74 b	0.73 b	0.58 d	0.64 c	0.84 a
Camphor	0.803 bc	1.033 a	0.72 c	0.856 b	0.72 c	0.843 b	0.783 bc
Borneol	0.05 bcd	0.07 b	0.096 a	0.06 bc	0.03 d	0.12 a	0.04 cd
α-cubebene	0.07 a	0.04bc	0.05 ab	0.04 bc	0.02 c	0.04 bc	0.05 ab
β-cubebene	0.226 a	0.213 ab	0.156 bc	0.223 a	0.013 d	0.196 abc	0.113 c
β-elemene	1.03 c	1.47 a	1.38 a	1.176 b	0.986 cd	1.23 b	0.87 d
t-muurolol	0.09 b	0.143 a	0.06 cd	0.08 bc	0.046 d	0.143 a	0.05 d
Cadina-1(10),4-diene	0.186 ab	0.11 c	0.21 a	0.176 ab	0.09 c	0.133 bc	0.08 c
Bornyl acetate	0.463 b	0.63 a	0.433 b	0.563 ab	0.486 b	0.65 a	0.296 c
Eugenol	4.62 c	8.44 a	6.24 b	4.55 c	5.61 bc	5.38 bc	5.43 bc
Methyleugenol	0.826 e	1.43 b	0.966 de	1.196 c	1.04 cd	1.79 a	1.016 d
Methylchavicol	0.11 e	1.41 a	1.36 a	0.59 c	0.39 d	0.14 e	0.99 b
Cubenol	0.55 b	0.713a	0.6 b	0.59 b	0.523 b	0.61 b	0.42 c
(-)-spathulenol	0.066 cb	0.086 ab	0.026 d	0.106 a	0.043 cd	0.113 a	0.04 cd
δ-cadinol	0.016 b	0.046 a	0.016 b	0.016 b	0.013 b	0.016 b	0.013 b
epi-α-cadinol	3.48 d	4.96 a	4.64 ab	4.24 cb	4.03 c	5.09 a	3.23 d

EO: essential oil, TN: untreated plants (normal condition), T10–12, T10–24, T10–48, T4–12, T4–24 and T4–48 refer to the cold-stressed plants cultivated under 12, 24 and 48 h at 4 and 10 °C. Common letters in each row show no significant difference at *P* ≤ 0.01.

### Influence of cold stress on genes expression level

**Figure 2:**
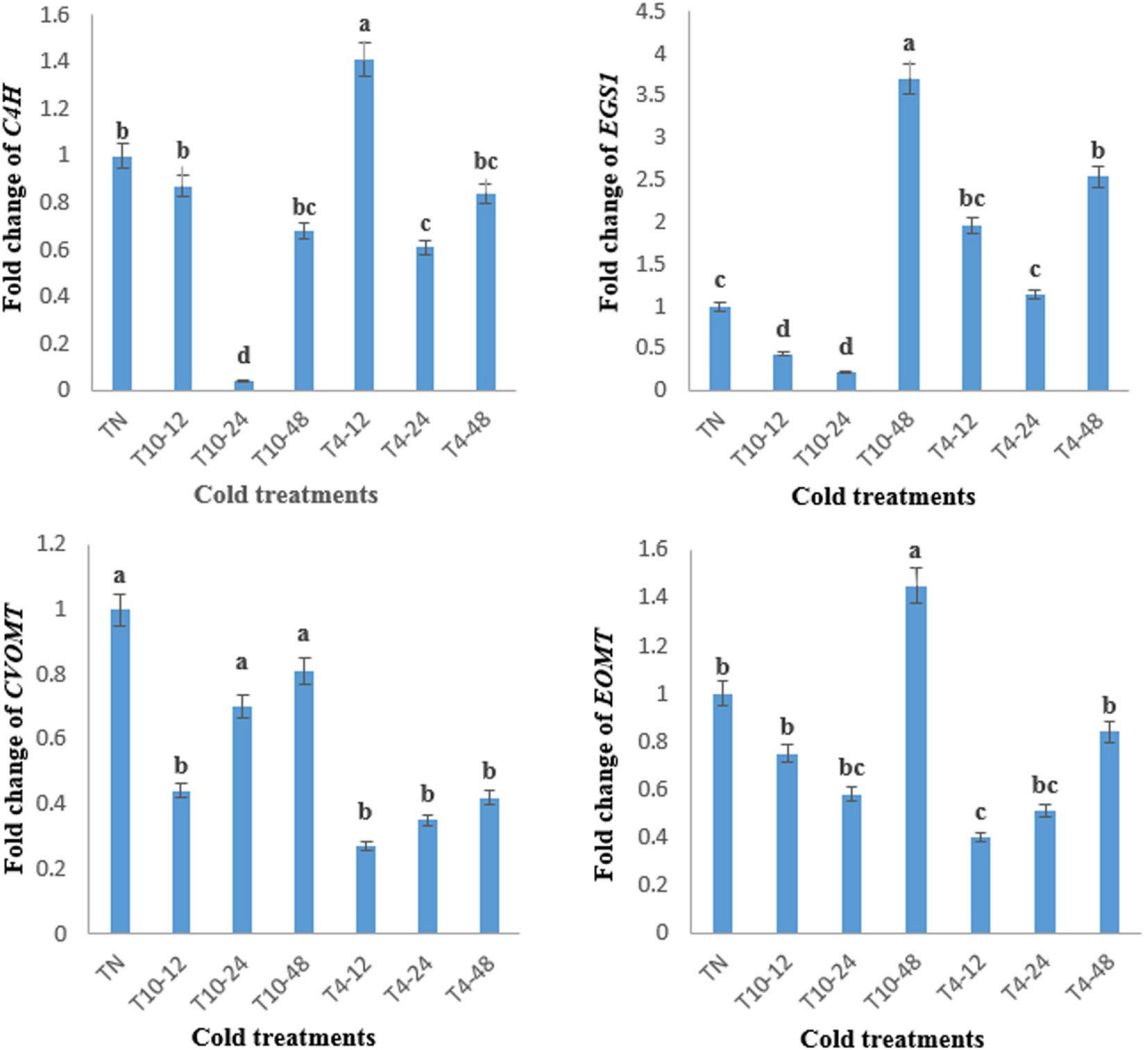
The expression levels of cinnamate 4-hydroxylase (*C4H*), eugenol synthase 1 (*EGS1*), chavicol *O*-methyl transferase (*CVOMT*) and eugenol *O*-methyl transferase (*EOMT*) genes in *Ocimum basilicum* under cold stress conditions. TN: untreated plants (normal condition), T10–12, T10–24, T10–48, T4–12, T4–24 and T4–48 refer to the cold-stressed plants cultivated under 12, 24 and 48 h at 4 and 10 °C. Common letters on the columns show no significant difference among the treatments at *P* ≤ 0.01.

The expression levels of *C4H*, *EGS1*, *EOMT* and *CVOMT* genes were assessed in plant leaves under cold stress conditions. Results revealed the significant (*P* ≤ 0.01) effect of cold stress on the expression levels of all genes, studied (Table 6). Temperature 10 °C for 48 h and 4 °C for 12, 24 and 48 h increased the expression level of *EGS1*. The expression level of *CVOMT* decreased by all cold treatments. The highest expression level of *EOMT* and *C4H* observed in T10–48 and T4–24, while the other level of treatments declined the expression of *EOMT* and *C4H* (Fig. 2).

**Table 6:** Analysis of variance for the expression of studied genes under cold stress in *Ocimum basilicum*.

Source of variance	Mean of squares			
	EGS1	EOMT	CVOMT	C4H
Cold stress	5.34**	0.42**	0.133**	0.59**
Error	0.25	0.32	0.011	0.017
Coefficient of variation **(%)**	30.07	23.63	21.43	17.53

*EGS1*: eugenol synthase1, *C4H*: cinnamate 4-hydroxylase, *EOMT*: eugenol *O*-methyl transferase, *CVOMT*: chavicole *O*-methyltransferase. **Means significant at level 0.01 (*P* ≤ 0.01).

## Discussion

Environmental stresses like cold may increase the cellular damage induced by the elevated ROS generation. Thus, cold stress resistance could be related, in part, to the enhancement of the antioxidative defense system, including antioxidant compounds (such as phenolic and flavonoids compounds) and several antioxidative enzymes. Phenolic compounds, the most widespread secondary metabolites in plants, can cleanse ROS and prevent lipid peroxidation, because of their strong ability to donate electrons and hydrogen atoms^30^. In our investigation, all cold stress treatments enhanced TPC over the control plants where the highest amount of that as wells as TFC were observed in T4–12. TPC significantly enhanced in lettuce leaf basil exposed to 6 °C^13^. Sivaci *et al*.^31^ demonstrated the association of plant response to stress stimuli with the levels of phenolic compounds. A substantial increments in soluble phenolic concentrations have been also indicated in tomato and watermelon plants grown in chilling conditions. The higher contents of phenolics in these plants have been explained by the enhanced activity of PAL^32^, which converts L-phenylalanine to ammonia and trans-cinnamic acid, a first step in the phenolic biosynthesis pathway. In our study, a positive significant association between TPC and PAL activity was detected.

The DPPH exhibits the activity of the non-enzymatic antioxidants (a part of antioxidant defense complex of plants). In our investigation, most of the cold treatments except T10–12 led to a DPPH expansion, which the highest level of that was obtained in T4–12. Kalisz *et al*.^10^ reported significant increases in DPPH in the red, lettuce leaf and cinnamon basil cultivars treated with 6 °C. They also reported that basil antioxidant properties (DPPH) are largely associated with TPC. Also, in the leaves of the lettuce plants exposed to various stresses, antioxidant capacity was positively associated with the total phenolic content^13^. In our study, a positive significant correlation was observed between TFC and DPPH. The correlation between TPC and DPPH was also positive but not significant. These findings suggests a possible major role of TFC in the detoxification of free radicals in basil plants grown in stress conditions.

The simultaneous activity of multiple antioxidant enzymes plays a vital role in protection of the plant cell against ROS toxicity^33^. In our study, the activity of SOD, CAT, APX and GPX enzymes were also investigated as a part of antioxidative defense system. All cold treatments enhanced the activity of SOD. The highest activity of SOD and GPX was achieved in T4–48, while this treatment caused the highest reduction in the activity of APX and CAT. Kalisz *et al*.^10^ reported a negligible alteration in CAT activity in all studied basil genotypes except Thai basil which showed a decline in CAT activity. They also stated the highest activity of peroxidases in Thai basil exposed to low temperature. Such differences in GPX and CAT enzyme activities observed in our study and Kalisz *et al*.’s investigation, might be caused by cellular location, an imbalance among antioxidant enzymes and CAT sensitivity to low temperature^30,33^. The observed decline in the CAT activity under T4–48 in our study could be compensated by an increase in the activity of other enzymes such as GPX^34^. Also, an alteration in the response of particular enzymes to the stressor, has been confirmed in several investigations. Lee and Lee (2000)^35^ noted for cucumber that chilling (4 °C for 12 h) enhanced the activities of SOD, APX, glutathione reductase (GR) and GPX, whereas it induced a decrease in CAT activity. Similarly, Gou *et al*.^36^ found an increase in peroxidase and APX activity in pepper seedlings subjected to chilling stress at 10/6 °C (day/night) but a decrease in CAT activity before 24 h.

Although the production of secondary metabolites in plants is controlled genetically, but environmental stresses and climate situations may affect their biosynthesis and variety in plants^18,37^. Changes in EOs percentage and composition has been investigated in medicinal plants under water stress^10^ but comprehensive information regarding the influence of cold stress on EO composition is limited. In our study, EO percentage and the content of phenylpropanoid compounds significantly affected by cold stress. The maximum percentage of EO was achieved in plants treated with 4 °C for 48 h. The amount of camphor, bornyl acetate, eugenol and methylchavicol was the highest in plants exposed to 10 °C for 12 h while the greatest amount of methyleugenol was obtained in plants treated with 4 °C for 24 h. An increase in eugenol and methylchavicol contents under different levels of irrigation has been indicated in basil^38^. Omidbaigi *et al*.^39^ indicated that the amount of methylchavicol and trans α-bergamotene enhance in sweet basil plants subjected to water deficit. Drought stress of 75% of field capacity (FC) increased EO percentage in both *O. basilicum* and *O. americanum*. Also eugenol and methyleugenol contents were increased in *O. basilicum* at 75% of FC but in *O. americanum*, drought stress reduced the amount of methyleugenol and iso-eugenol. An increase in carvacrol content in *Satureja hortensis* L. under limited water stress has been already reported, while terpinene content decreased under limited and intense water stress in this plant^40^. However, environmental stresses might affect the EO and its constituents through the influence on the transcription of the genes and activity of the enzymes involved in their biosynthesis^18,41^.

Secondary metabolites like phenylpropanoid compounds protect the plants against the biotic and abiotic stresses. Rapid induction of the transcription of the genes responsible for the production of secondary metabolites under environmental stresses may elevate the accumulation of these metabolites. In other words, stress leads to activate the plant defense strategies and production of secondary metabolites^30,42^. Recent investigations have confirmed the production of secondary metabolites, gene expression and post-translational modifications as key responses of the plants against environmental stresses^18,43^. Among the stresses, cold affect the plant life metabolisms^44^. Hence, to partially unravel the mechanism by which phenylpropanoid content changed in *O. basilicum* under cold stress, the expression levels of *C4H*, *EGS1*, *EOMT* and *CVOMT* genes were measured in our investigation. C4H enzyme converts cinnamate to 4-hydroxy-cinnamate, an important reaction of the phenylpropanoid pathway which ends with the biosynthesis of several secondary metabolites. EGS1 converts substrate coniferyl acetate to eugenol. At the end of this pathway, OH-4 of eugenol and chavicol are methylated respectively by eugenol *O*-methyl transferase (EOMT) and chavicol *O*-methyl transferase (CVOMT) enzymes and methyleugenol and methylchavicol are produced^7,8^. The genes expression levels mentioned above have not been examined in *O. basilicum* plants treated with cold stress but it has been already demonstrated that chitosan and salicylic acid increase the *CVOMT* expression in basil^37,43^. The expression of *C4H* decreased in response to drought stress, abscisic (ABA) and giberellic (GA3) acids but increased in response to wounding in tea^45^. In our study, moderate cold stress (10 °C) for 48 h upregulated the expression levels of *EGS1* and *EOMT* but that of *CVOMT* relatively remained constant. The eugenol content was relatively unchanged but those of methyleugenol and methylchavicol were increased under this treatment compared to the controls. The highest expression level of *C4H* was obtained in 4 °C for 12 h. The content of eugenol, methyleugenol and methylchavicol was also increased under this treatment. This congruence between *C4H*, *EGS1* and *EOMT* expression and eugenol, methyleugenol and methylchavicol contents might be due to the presence of cold responsive elements in the promoters of these genes^18^. Also, *C4H, EGS1* and *EOMT* genes could be suggested as potential candidate genes for genetically manipulation of the phenylpropanoid biosynthesis pathway for enhancement of the valuable EO constituents in basil.

## Concluding remarks

A positive significant association observed between TFC and DPPH in our study, suggests TFC as a major free radicals scavenger in basil plants grown under cold stress. Moreover, the enhancement of SOD activity confirms its crucial role in scavenging superoxide radicals produced in basil plants grown under cold stress. The lowest activity of APX, CAT and PAL enzymes observed in severe cold stress (T4–48), demonstrated the sensitivity of these enzymes to low temperature. The content of eugenol, methyleugenol and methylchavicol was positively associated with the expression level of *EGS1* and *EOMT* genes under 10 °C for 48 h. The expression of *C4H* upregulated by 4 °C for 12 h; the content of three above-mentioned compounds was also increased under this treatment. The observed consistency between *C4H*, *EGS1* and *EOMT* expressions and the contents of eugenol, methyleugenol and methylchavicol suggests that cold stress probably increases the content of these compounds, partially, via upregulating the expression of *C4H*, *EGS1* and *EOMT* but further research such as determining the activity of the encoded enzymes by these genes and expression of the other genes responsible for phenylpropanoid production is required to more clarify the mechanism of phenylpropanoid regulation under cold stress. Our results also proposes the exposure of basil seedlings to the temperature 10 °C or below as an easy technique for raising the content of important EO compounds. In Iran, basil is cultivated in spring in warm provinces such as Khuzestan (in the south of Iran), hence early planting of the basil in spring or at the end of winter may also enhance the EO compounds.

## Supplementary information

Supplementary information.

## Data Availability

All the materials used and data produced in our experiment will be available for the readers.
