# Supplementary material for: Cold stress changes antioxidant defense system, phenylpropanoid contents and expression of genes involved in their biosynthesis in Ocimum basilicum L
Source: Sci Rep. 2020 Mar 24;10:5290. doi: 10.1038/s41598-020-62090-z (PMC7093387; doi:10.1038/s41598-020-62090-z)

Cold stress changes antioxidant defense system, phenylpropanoid contents and expression of genes involved in their biosynthesis in *Ocimum basilicum* L.

Ramin Rezaie [a,b], Babak Abdollahi Mandoulakani [ab*], Mohammad Fattahi [c]

[a] Department of Plant Production and Generics, Faculty of Agriculture, Urmia University, Urmia, Iran.

[b] Department of Agricultural Biotechnology, Institute of Biotechnology, Urmia University, Urmia, Iran

[c] Department of Horticultural Sciences, Faculty of Agriculture, Urmia University, Urmia, Iran.

* Corresponding author

E-mail address: b.abdollahi@urmia.ac.ir

Tel: 00989122386990 Fax: 00984432779558

Supplementary Table S1

Essential oil compounds identified in *Ocimum basilicum* under cold stress using Gas chromatography/mass spectrometry (GC/MS)

	Compounds	Empirical formula	Retention time	RI	Identification methods
1	α-pinene	C10H16	10.73	935	GC-MS, IR, Ref
2	Camphene	C10H16	11.37	945	GC-MS, IR, Ref
3	(-)-β-pinene	C10H16	12.67	965	GC-MS, IR, Ref
4	β -myrcene	C10H16	13.58	977	GC-MS, IR, Ref
5	1,8-cineole	C10H18O	15.32	1000	GC-MS, IR, Ref
6	(E)-β-ocimene	C10H16	16.34	1037	GC-MS, IR, Ref
7	cis- β-terpineol	C10H18O	17.3	1052	GC-MS, IR, Ref
8	Terpinolene	C10H16	18.15	1075	GC-MS, IR, Ref
9	Linalool	C10H18O	19.38	1114	GC-MS, IR, Ref
10	Camphor	C10H16O	20.89	1147	GC-MS, IR, Ref
11	Borneol	C10H18O	22.09	1170	GC-MS, IR, Ref
12	α-terpineol	C10H18O	23.47	1172	GC-MS, IR, Ref
13	Terpinen-4-ol	C10H18O	22.65	1181	GC-MS, IR, Ref
14	α-terpineol	C10H18O	23.63	1196	GC-MS, IR, Ref
15	Methylchavicol	C10H12O	23.87	1205	GC-MS, IR, Ref
16	Geraniol	C10H18O	24.33	1271	GC-MS, IR, Ref
17	Bornyl acetate	C12H20O2	27.46	1296	GC-MS, IR, Ref
18	α-cubebene	C15H24	30.36	1356	GC-MS, IR, Ref
19	Eugenol	C10H12O2	31.45	1378	GC-MS, IR, Ref
20	β-cubebene	C12H20O2	32.7	1390	GC-MS, IR, Ref
21	β-elemene	C15H24	32.89	1395	GC-MS, IR, Ref
22	Methyleugenol	C11H14O2	34.47	1419	GC-MS, IR, Ref
23	α-bergamotene	C15H24	35.52	1446	GC-MS, IR, Ref
24	α-guaiene	C15H24	35.63	1450	GC-MS, IR, Ref
25	α-caryophyllene	C15H24	36.31	1478	GC-MS, IR, Ref
26	E-β-farnesene	C15H24	36.71	1451	GC-MS, IR, Ref
27	epi-bicyclosesquiphellandrene	C15H24	36.8	1474	GC-MS, IR, Ref
28	germacrene D	C15H24	37.64	1495	GC-MS, IR, Ref

29	(Z)-β-farnesene	C15H24	37.83	1502	GC-MS, IR, Ref
30	γ-elemene	C15H24	38.29	1509	GC-MS, IR, Ref
31	β-guaiene	C15H24	38.68	1518	GC-MS, IR, Ref
32	γ-cadinene	C15H24	39.04	1527	GC-MS, IR, Ref
33	Cadina-1(10),4-diene	C15H24	39.39	1535	GC-MS, IR, Ref
34	α-sesquiphellandrene	C15H26O	39.5	1539	GC-MS, IR, Ref
35	unknown	C15H24	39.77	1549	GC-MS, IR
36	δ-cadinol	C15H26O	40.37	1558	GC-MS, IR, Ref
37	(-)-spathulenol	C15H26O	41.41	1583	GC-MS, IR, Ref
38	Cubenol	C15H24O	42.55	1618	GC-MS, IR, Ref
39	epi-α-cadinol	C15H26O	43.38	1647	GC-MS, IR, Ref
40	t-muurolol	C15H26O	43.8	1663	GC-MS, IR

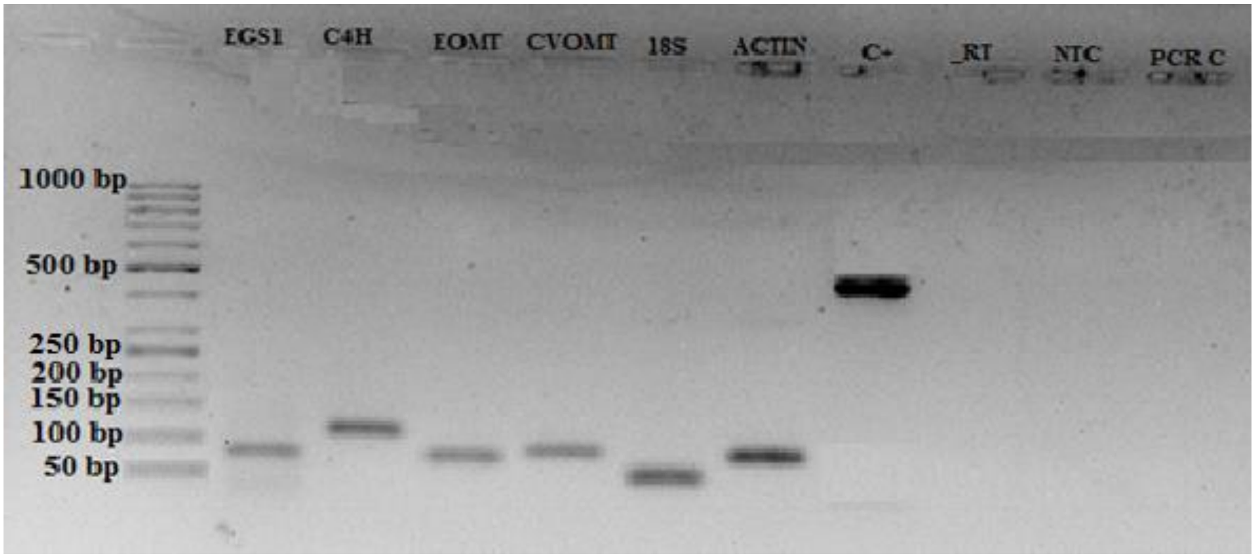

Supplementary Figure 1: Gene fragments amplified from cDNAs by primers of genes: eugenol synthase1 (*EGS1*), cinnamate 4-hydroxylase (*C4H*), eugenol *O*-methyl transferase (*EOMT*), chavicole *O*-methyltransferase (*CVOMT*), 18srRNA, actin, C^{+}: amplification of glyceraldehyde 3-phosphate dehydrogenase (*GAPDH*) gene from cDNA synthesized from control RNA (1.3kb), supplied with the kit RevertAid™ First Strand cDNA Synthesis Kit (Thermo Scientific, USA) according to the manufacturer's instructions, -RT: no reverse transcriptase during cDNA synthesis, NTC: negative template control during cDNA synthesis, PCR C: PCR control. This cropped gel has been prepared by Snagit 10 Editor software and obtained from original gels with different running times (as below) which is captured using a Gel documentation system (Infinity, France).

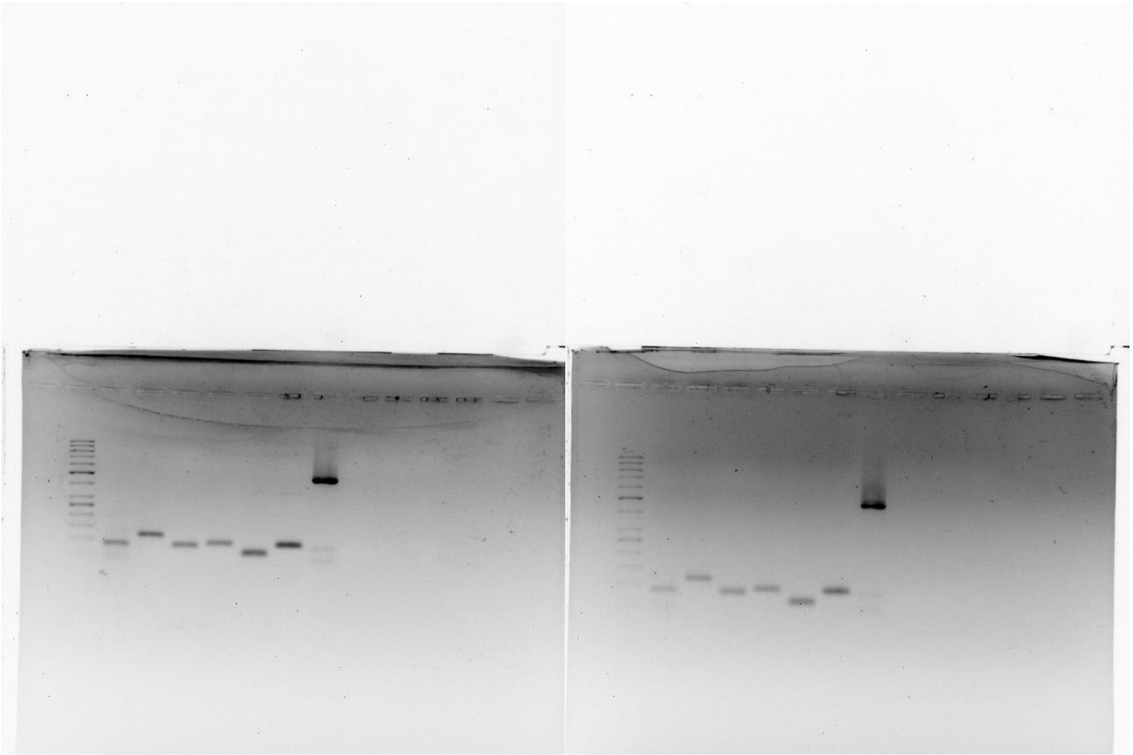

Supplement: Supplementary file 1 — Supplementary information. [file 41598_2020_62090_MOESM1_ESM.pdf]
